# An integrated modeling approach for estimating monthly global rainfall erosivity

**DOI:** 10.1038/s41598-024-59019-1

**Published:** 2024-04-08

**Authors:** Ayele A. Fenta, Atsushi Tsunekawa, Nigussie Haregeweyn, Hiroshi Yasuda, Mitsuru Tsubo, Pasquale Borrelli, Takayuki Kawai, Ashebir S. Belay, Kindiye Ebabu, Mulatu L. Berihun, Dagnenet Sultan, Tadesual A. Setargie, Abdelrazek Elnashar, Arfan Arshad, Panos Panagos

**Affiliations:** 1 International Platform for Dryland Research and Education, Tottori University, Tottori, 680-0001 Japan; 2 Arid Land Research Center, Tottori University, 1390 Hamasaka, Tottori, 680-0001 Japan; 3 Organization for Educational Support and International Affairs, Tottori University, Koyama Minami 4-101, Tottori, 680-8550 Japan; 4 Department of Environmental Sciences, University of Basel, 4056 Basel, Switzerland; 5 Department of Science, Roma Tre University, Rome, Italy; 6 Graduate School of International Resource Sciences, Akita University, 1-1 Tegatagakuen-Machi, Akita, 010-8502 Japan; 7 Department of Earth Science, Bahir Dar University, P.O. Box 79, Bahir Dar, Ethiopia; 8 College of Agriculture and Environmental Sciences, Bahir Dar University, P.O. Box 1289, Bahir Dar, Ethiopia; 9 Faculty of Civil and Water Resource Engineering, Bahir Dar Institute of Technology, Bahir Dar University, P.O. Box 26, Bahir Dar, Ethiopia; 10 Tropical Research and Education Center, University of Florida, Gainesville, FL 33031 USA; 11 Department of Natural Resources, Faculty of African Postgraduate Studies, Cairo University, Giza, 12613 Egypt; 12 Department of Biosystems and Agricultural Engineering, Oklahoma State University, Stillwater, OK 74075 USA; 13 European Commission, Joint Research Centre (JRC), 21027 Ispra, VA Italy

**Keywords:** Environmental sciences, Hydrology

## Abstract

Modeling monthly rainfall erosivity is vital to the optimization of measures to control soil erosion. Rain gauge data combined with satellite observations can aid in enhancing rainfall erosivity estimations. Here, we presented a framework which utilized Geographically Weighted Regression approach to model global monthly rainfall erosivity. The framework integrates long-term (2001–2020) mean annual rainfall erosivity estimates from IMERG (Global Precipitation Measurement (GPM) mission’s Integrated Multi-satellitE Retrievals for GPM) with station data from GloREDa (Global Rainfall Erosivity Database, *n* = 3,286 stations). The merged mean annual rainfall erosivity was disaggregated into mean monthly values based on monthly rainfall erosivity fractions derived from the original IMERG data. Global mean monthly rainfall erosivity was distinctly seasonal; erosivity peaked at ~ 200 MJ mm ha^−1^ h^−1^ month^−1^ in June–August over the Northern Hemisphere and ~ 700 MJ mm ha^−1^ h^−1^ month^−1^ in December–February over the Southern Hemisphere, contributing to over 60% of the annual rainfall erosivity over large areas in each hemisphere. Rainfall erosivity was ~ 4 times higher during the most erosive months than the least erosive months (December–February and June–August in the Northern and Southern Hemisphere, respectively). The latitudinal distributions of monthly and seasonal rainfall erosivity were highly heterogeneous, with the tropics showing the greatest erosivity. The intra-annual variability of monthly rainfall erosivity was particularly high within 10–30° latitude in both hemispheres. The monthly rainfall erosivity maps can be used for improving spatiotemporal modeling of soil erosion and planning of soil conservation measures.

## Introduction

Soil erosion by water is a global environmental threat^1,2^ that adversely impacts ecosystem services^3–5^. Climate change and the concomitant increase in rainfall erosivity are expected to affect more than 85% of the Earth^6^, which will exacerbate future environmental degradation caused by soil erosion^7^. Combating land degradation by soil erosion has been a key focus of efforts to accomplish several of the United Nations' Sustainable Development Goals (SDGs^8^) and raised interest in investigating soil erosion at regional and global scales. However, because the high spatial heterogeneity of erosion-controlling factors makes investigating large-scale soil erosion impossible, studies are frequently conducted at small scale. Large-scale soil erosion studies have therefore relied mainly on models. The Revised Universal Soil Loss Equation (RUSLE^9^) is commonly used to simulate annual soil erosion rates at regional and global scales^10–15^. Because rates of soil erosion depend on, inter alia, the seasonality of rainfall and vegetation cover^9,16^, modeling soil erosion at higher temporal resolutions (e.g., monthly) is needed to improve soil erosion predictions and conservation planning efforts.

Among the factors that cause soil erosion by water, rainfall erosivity is the most dynamic on an intra-annual basis^17^. In the RUSLE model, rainfall erosivity represents the triggering of soil loss by sheet and rill erosion processes during rainfall events^16^ and is not controlled by human actions, unlike land-cover management factors. The monthly distribution of rainfall erosivity impacts water management, agricultural practices^16^, and land cover by protective vegetation^18^. The high intensity rainfall events in certain months of the year can account for the highest proportion of annual soil loss^19^. Monthly rainfall erosivity data are thus critical for informing decisions about crop and tillage practices, particularly during months of high rainfall erosivity^20^. Modeling efforts can be used to identify months and regions potentially subject to high rainfall erosivity, i.e., when and where priority remedial measures should be implemented. Monthly rainfall erosivity has mainly been studied in Europe, where a relatively dense network of ground-based gauges allowed for recording of extreme rainfall events with a higher temporal frequency^17,19–22^. However, many critical regions across the world have gone unstudied due to sparse distributions of rainfall monitoring stations.

Computing rainfall erosivity requires long-term rainfall data at sub-hourly intervals^9,16^. Globally, rain gauges that measure rainfall at short time intervals (e.g., 30 min) are limited in terms of spatiotemporal coverage, especially in Africa, South America, and Asia^23^. Previous studies employed interpolation of gauge data to provide distributed estimates of rainfall erosivity in non-monitored areas^17,19,22–24^. However, interpolation of point data can lead to large uncertainties in regions with few gauging stations^25^. Recent advances in remote sensing allow for providing high-resolution rainfall estimates with global coverage^26,27^, and have been widely applied to assess rainfall erosivity in various regions including China^28^, India^29^, USA^30^, Eastern Africa^31^, and many other global regions^32^. Nevertheless, errors in retrieval algorithms, sampling frequency, and other factors in satellite observations can lead to uncertainties in rainfall erosivity estimations^33–35^. Integrating rainfall data from satellites and ground-based stations can benefit from the spatial coverage of satellite observations and the accuracy of gauge measurements^36^. Recent studies have also demonstrated that integrating satellite and gauge data markedly enhances the accuracy of estimated rainfall erosivities^37^.

Our paper advances the estimation of intra-annual variability of global rainfall erosivity by integrating IMERG (Global Precipitation Measurement (GPM) missions’ Integrated Multi-satellitE Retrievals for GPM^26^ data with gauge data from GloREDa (Global Rainfall Erosivity Database^23^. Specifically, we aimed to (i) model global monthly and seasonal rainfall erosivities, (ii) investigate their latitudinal distributions, and (iii) develop global maps of the months of maximum rainfall erosivity and erosivity density. We analyzed long-term (2001–2020) mean annual global rainfall erosivity based on the IMERG rainfall dataset at 30-min temporal and 0.1° × 0.1° spatial resolution. We then integrated IMERG’s mean annual rainfall erosivity estimates and GloREDa data^23^ using Geographically Weighted Regression (GWR^36^). The merged mean annual rainfall erosivity was then temporally disaggregated into monthly values using monthly rainfall erosivity fractions computed from the original IMERG data. Findings from this study can assist in improving our understanding of the global distribution of monthly and seasonal rainfall erosivities and facilitate soil erosion modeling and soil conservation planning.

## Results and discussion

### Evaluation of rainfall erosivity estimated by IMERG merged with GloREDa

Cross-validation of the mean annual rainfall erosivity estimated by integrating IMERG and GloREDa (Fig. S1a, b) revealed reasonably good performance^37^. Fenta et al.^37^ demonstrated that the accuracy of mean annual rainfall erosivity estimates markedly improved by GWR-based integration of IMERG and GloREDa. Our results of the merged mean annual erosivity estimates had lower PBIAS (− 2.4%), higher NSE (0.83), and lower RMSE (1122 MJ mm ha^−1^ h^−1^ yr^−1^) compared to those estimated from IMERG data alone (PBIAS =  + 27.8%, NSE = 0.51, and RMSE = 1730 MJ mm ha^−1^ h^−1^ yr^−1^). The accuracy of GWR-based merging of rainfall erosivity was relatively better for the regions with higher rain gauges density^37^. Detailed cross-scale evaluation of the IMERG-based mean annual rainfall erosivity estimates has been provided by Fenta et al.^37^. Gauges.

**Figure 1:**
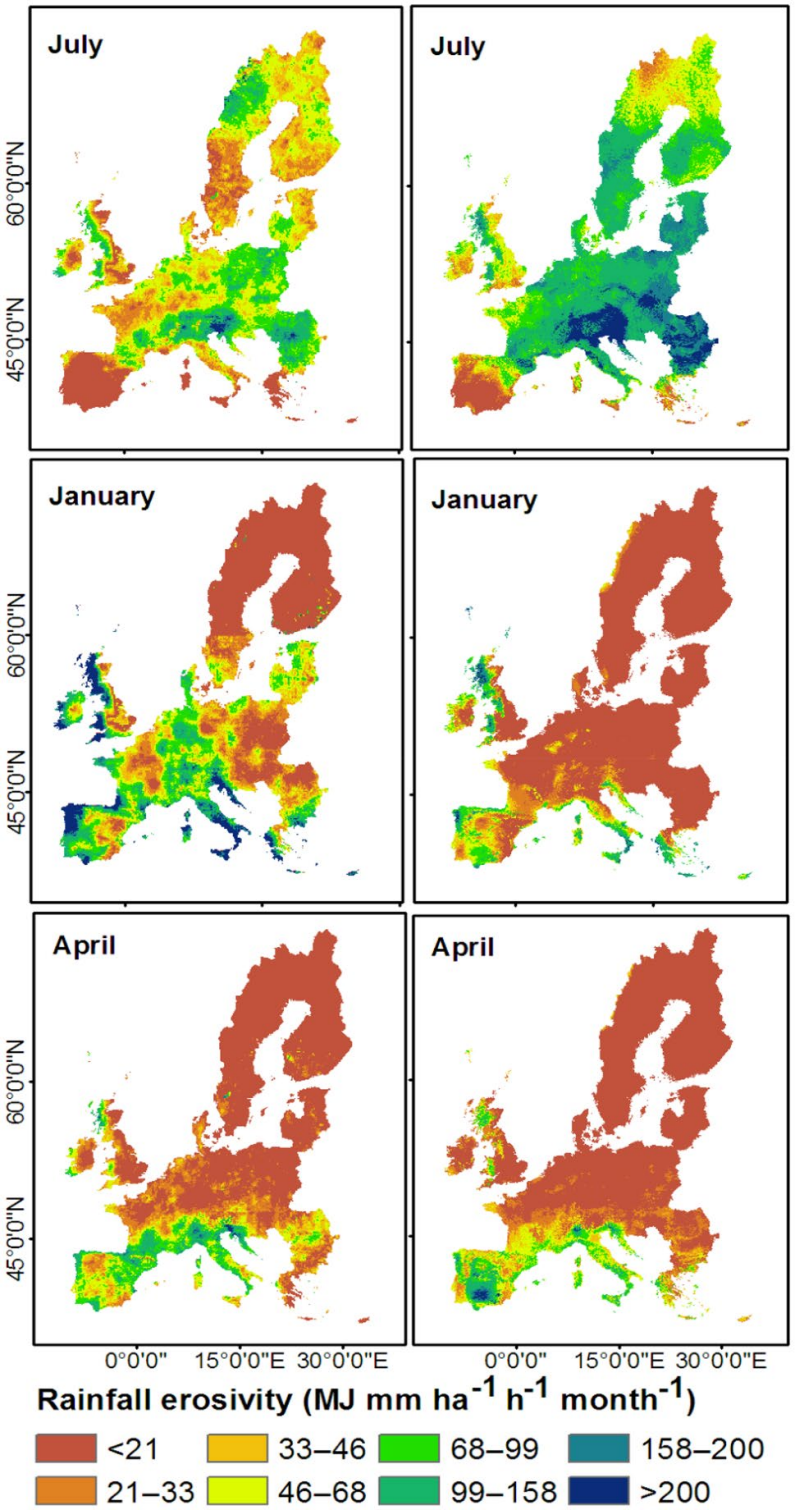
Comparison between monthly erosivities estimated for Europe based on Integrated Multi-satellitE Retrievals for Global Precipitation Measurement (IMERG) merged with Global Rainfall Erosivity Database (GloREDa) (left panel) and interpolated from GloREDa data alone (Ballabio et al.^17^; right panel). This map was produced using ArcGIS Pro version 3.2 (https://www.esrij.com/products/arcgis-pro/).

We compared the merged monthly rainfall erosivities for Europe with the monthly erosivity maps of Ballabio et al.^17^. Figure 1 shows monthly erosivity values for three selected months in which the merged estimates were underestimated (July), overestimated (January), or in good agreement (April) with those interpolated from GloREDa^17^. The merged estimates markedly underestimated rainfall erosivities in July over the Alpine region and Eastern Europe (Fig. 1). Similarly, Bezak et al.^32^ found underestimations of rainfall erosivity by satellite-based estimates over the Alpine region of Europe for the month of July. In January, the merged estimates largely overestimated rainfall erosivities over western Europe and the Alpine region (Fig. 1). The best agreement between merged monthly erosivity estimates and GloREDa-interpolated values over large parts of Europe was observed in April (Fig. 1). Seasonal comparisons (Fig. S2) showed that the merged rainfall erosivity estimates agreed well with GloREDa-interpolated values over large parts of Europe during March–May and September–November, but underestimated seasonal rainfall erosivity over the Alpine region during June–August and overestimated seasonal erosivity over the Italian peninsula and western Europe during December–February (Fig. S2). Underestimates and overestimates of rainfall erosivity were likely due to the underestimation of high intensity rainfall events and the overestimation of low intensity rainfall events, respectively^33,35,37^.

### Spatial distribution of monthly rainfall erosivity

**Figure 2:**
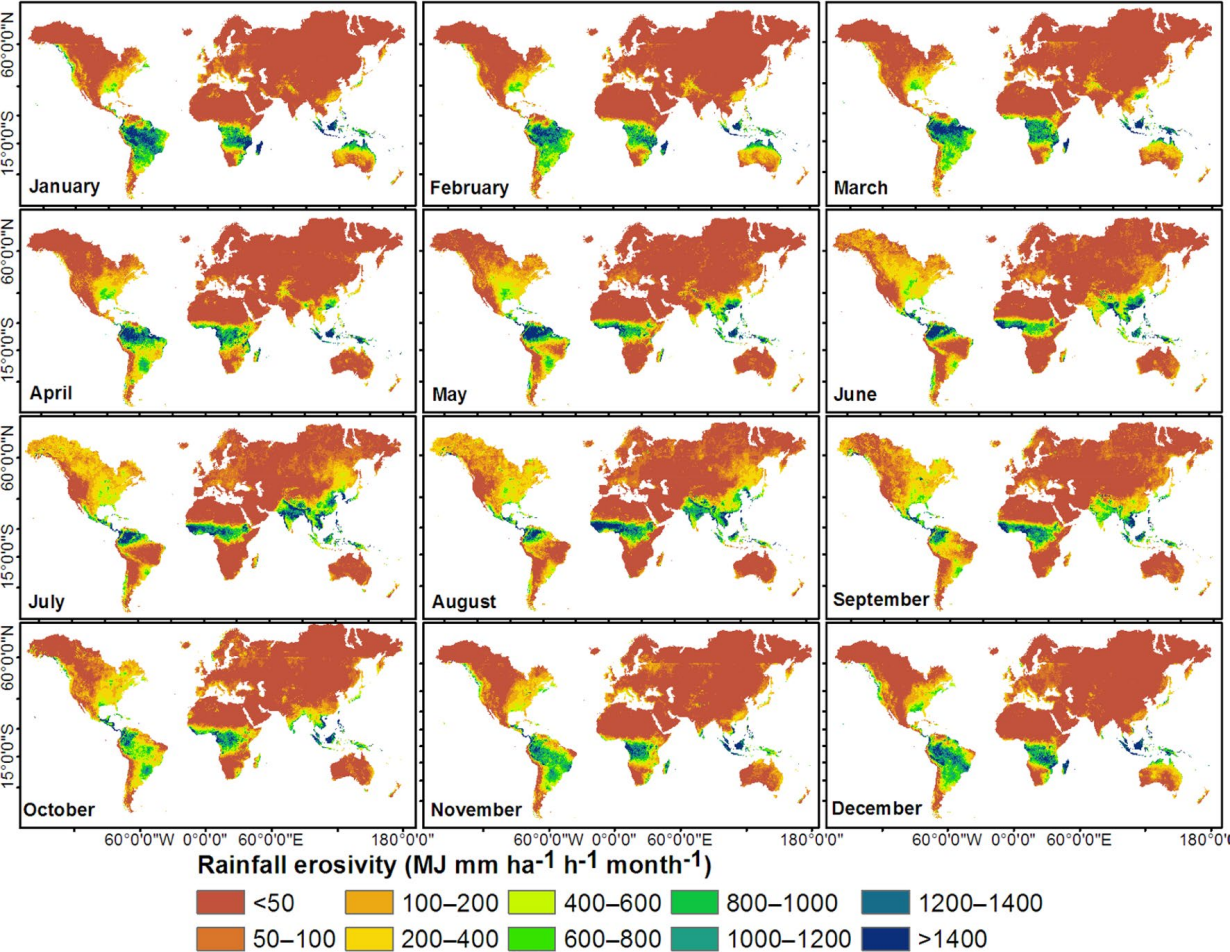
Spatial distribution of monthly rainfall erosivity based on temporally disaggregated mean annual estimates based on Integrated Multi-satellitE Retrievals for Global Precipitation Measurement (IMERG) merged with Global Rainfall Erosivity Database (GloREDa). This map was produced using ArcGIS Pro version 3.2 (https://www.esrij.com/products/arcgis-pro/).

The monthly rainfall erosivity maps (Fig. 2) revealed a gradient from higher erosivities in the tropical regions to lower erosivities towards sub-tropical (temperate) and polar regions. Equatorial South America, Africa, and Asia are subject to high rainfall erosivity (> 1,000 MJ mm ha^−1^ h^−1^ month^−1^, Fig. 2) for more than half of the year. The tropical north and south regions experienced high monthly rainfall erosivity during June–September and December–March, respectively (Fig. 2, Table 1). The highest monthly rainfall erosivities were observed in July and August (~ 700 MJ mm ha^−1^ h^−1^ month^−1^) in the tropical north and in January (1,045 MJ mm ha^−1^ h^−1^ month^−1^) in the tropical south (Table 1). The higher monthly rainfall erosivities in the Southern Hemisphere than in the Northern Hemisphere (Table 1) are likely attributable to the effects of averaging over a larger area in the Northern Hemisphere. The highest global mean monthly rainfall erosivity was in July and August (~ 210 MJ mm ha^−1^ h^−1^ month^−1^), whereas the lowest value was in November (146 MJ mm ha^−1^ h^−1^ month^−1^) (Table 1). The pattern of monthly rainfall erosivity (Fig. 2) followed the typical seasonality of rainfall^38,39^: high monthly mean daily rainfall values were observed in July and August globally and in the Northern Hemisphere and in January and February in the Southern Hemisphere. Zipser et al.^40^ also reported high-intensity storms, notably in June–August (Northern Hemisphere) and December–February and March–May (Southern Hemisphere).

**Table 1:** Distribution of mean monthly rainfall erosivity (MJ mm ha^−1^ h^−1^ month^−1^).

Month	Tropical north	Tropical south	Northern Hemisphere	Southern Hemisphere	Global
January	145	**1045**	54	**757**	175
February	125	919	47	674	155
March	204	960	64	694	173
April	313	649	84	476	152
May	515	404	131	309	161
June	601	221	176	188	177
July	**704**	172	**223**	148	**210**
August	**742**	154	**217**	132	**205**
September	613	231	177	185	178
October	442	406	117	319	152
November	261	634	78	474	146
December	191	844	66	615	161

High values are shown in bold font.

**Figure 3:**
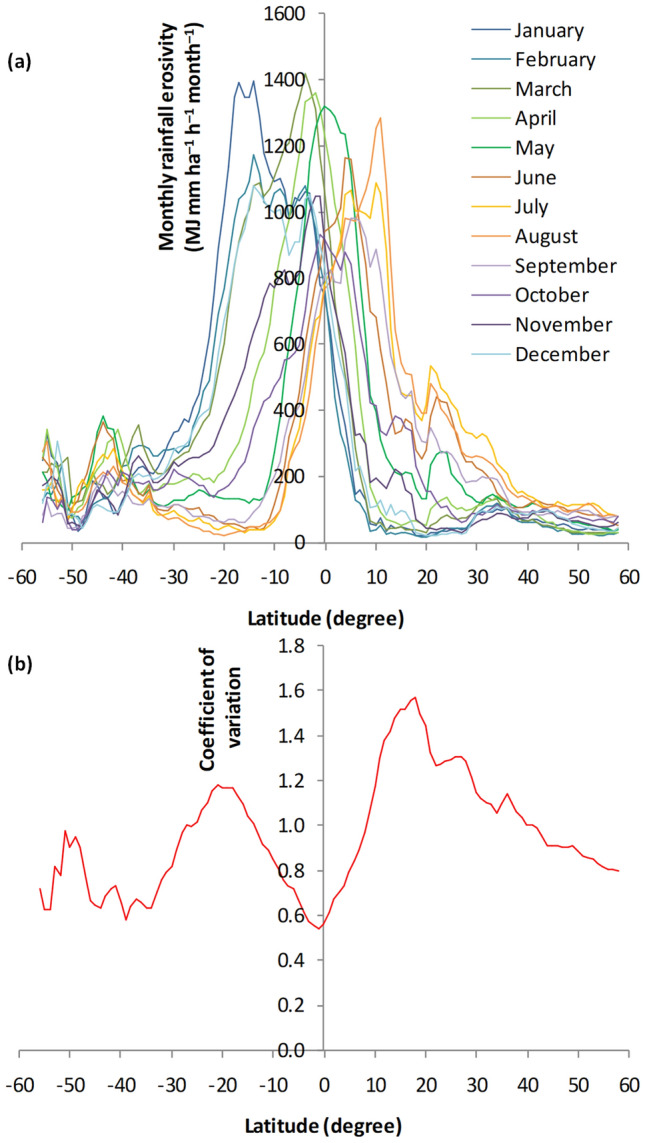
Latitudinal distribution of (**a**) monthly rainfall erosivity and (**b**) its coefficient of variation computed based on zonal averages at one-degree intervals between 60°S (–60°) and 60°N (60°).

The latitudinal profile of a zonally averaged monthly rainfall erosivity (Fig. 3a) provides a near-global view of the intra-annual variability. Figure 3a shows that rainfall erosivity values were heterogeneous across latitudes, with maxima occurring in tropical regions. Equatorial regions (e.g., northern South America, central Africa, and southeast Asia; Fig. 2) were likely to have high rainfall erosivities (> 1,000 MJ mm ha^−1^ h^−1^ month^−1^) for large parts of the year, with maxima occurring from March to May (Fig. 3a). The northern tropics and subtropics experienced high rainfall erosivity from June to September, whereas the tropical south experienced high rainfall erosivity from December to March (Fig. 3a). Subtropical (temperate) regions experienced relatively low rainfall erosivity (< 500 MJ mm ha^−1^ h^−1^ month^−1^, Fig. 3a). The latitudinal distribution of monthly rainfall erosivity followed the pattern of rain bands associated with the north–south migration of the Intertropical Convergence Zone^41^; relatively high monthly mean daily rainfall occurred in tropical regions throughout the globe^42,43^. Figure 3b shows the latitudinal distribution of the coefficient of variation (CV) of the monthly rainfall erosivity. CV values less than 1 indicate an even distribution of rainfall erosivity throughout the year, whereas values greater than 1 indicate a more heterogeneous distribution during the year (Fig. S3). Equatorial and subtropical regions in the north and south experienced a relatively even distribution of rainfall erosivity throughout the year (Fig. 3b). Intra-annual variability of monthly rainfall erosivity was particularly high for areas between 10 and 30°N and S (Fig. 3b); this pattern is likely attributable to the highly variable monthly rainfall erosivity in the region (Fig. 3a).

### Identification of months with maximum rainfall erosivity

**Figure 4:**
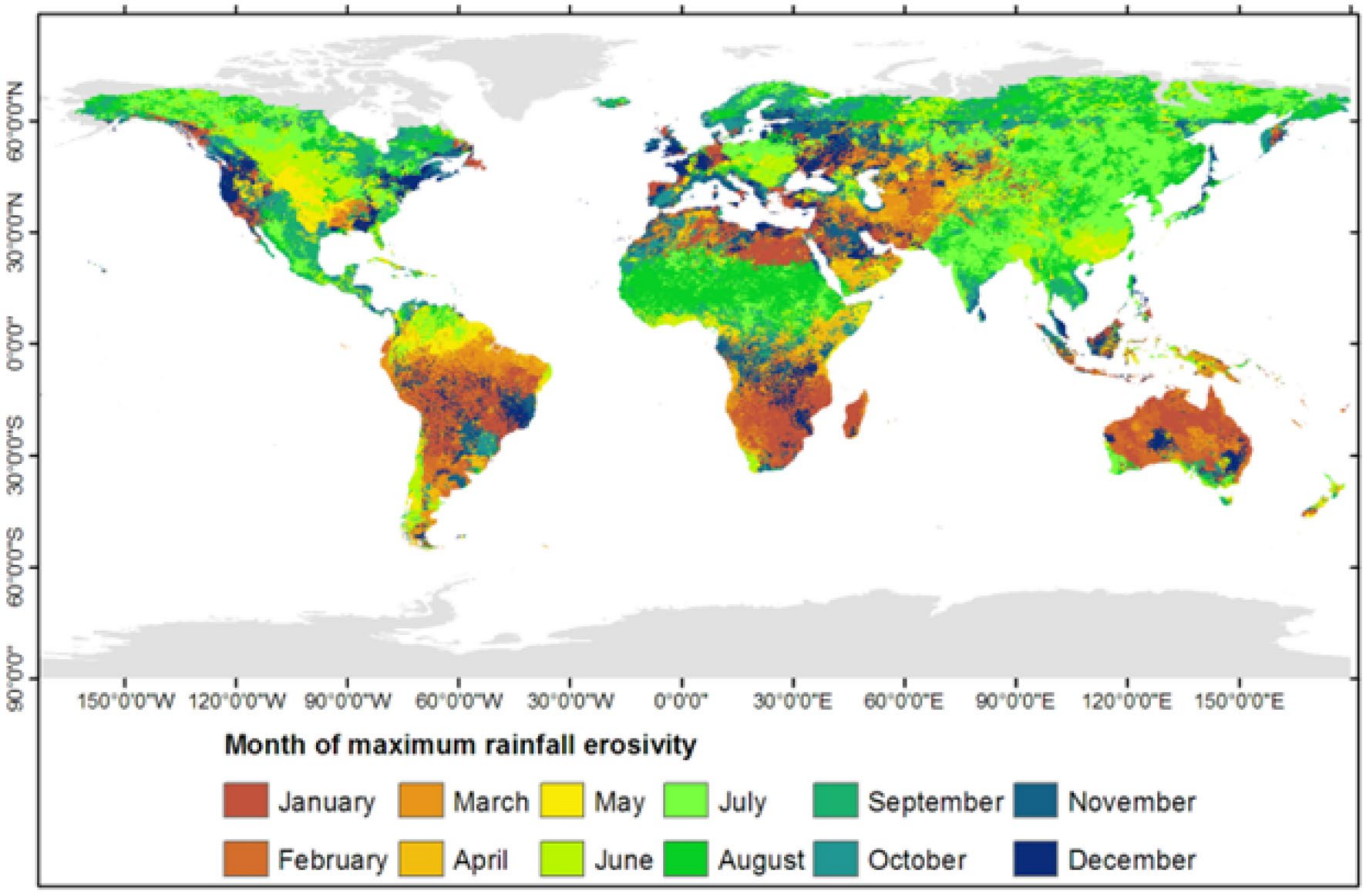
Map of the month of the year with the highest monthly rainfall erosivity value. This map was produced using ArcGIS Pro version 3.2 (https://www.esrij.com/products/arcgis-pro/).

Mapping monthly rainfall erosivity allows identification of the month with the highest rainfall erosivity within the year (Fig. 4). The severity of soil erosion depends on both the magnitude of rainfall erosivity and the time of the year when maximum rainfall erosivity occurs. This dual dependence mainly reflects the fact that other factors that change during the year, such as crop cover in croplands and vegetation conditions in non-croplands, also control soil erosion. Large parts of South America, southern Africa, and Australia experience maximum rainfall erosivity during the months of January and February (Fig. 4). Sizable parts of northern Africa and northern Europe have the highest rainfall erosivity in August, whereas July is the month of maximum rainfall erosivity in central Europe and large parts of Asia and northern North America (Fig. 4). Central North America experiences maximum rainfall erosivity in May and June (Fig. 4). October and November are the months of maximum rainfall erosivity for large parts of the Mediterranean region (Fig. 4). Identifying the month of the year when maximum rainfall erosivity occurs is crucial for optimizing measures to control soil erosion. Such knowledge can assist in reducing the risk of soil erosion by applying proper farming and management techniques^17,20,21^. For instance, in regions with high rainfall erosivity, the selection of appropriate tillage practices (e.g., minimum tillage), crop varieties, and cover crops is crucial to minimizing the risk of soil erosion during the most erosive months^21^. Proper management of grazing land such as rotational grazing or establishment of exclosures^44,45^ can help prevent overgrazing and thus subsequent soil erosion during the months of high rainfall erosivity.

### Monthly erosivity density

**Figure 5:**
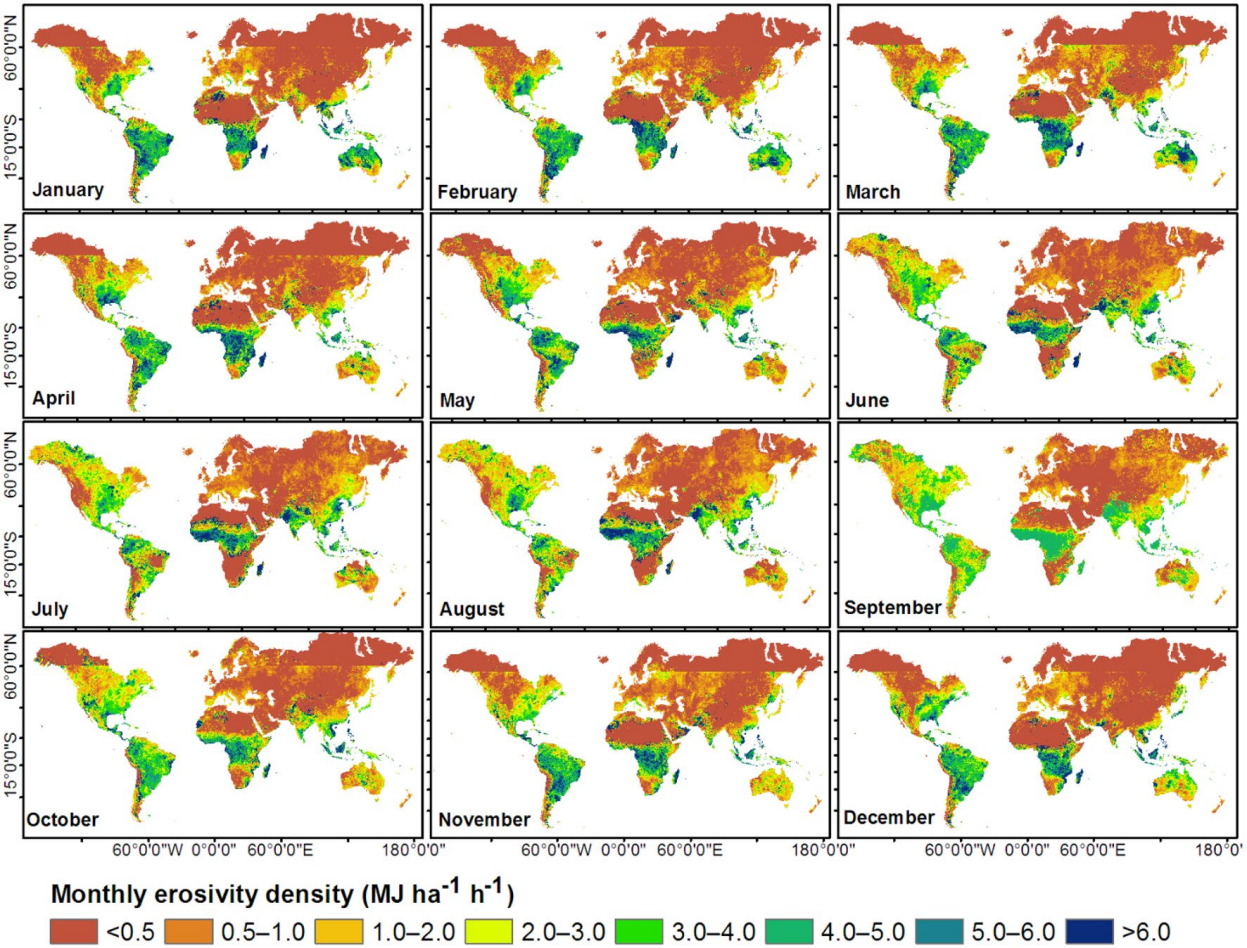
Spatial distribution of monthly erosivity density computed based on monthly rainfall erosivity (Fig. 2) and mean monthly rainfall from the Climatologies at High resolution for Earth’s Land Surface Areas (CHELSA) dataset. This map was produced using ArcGIS Pro version 3.2 (https://www.esrij.com/products/arcgis-pro/).

Examination of monthly EDs (Fig. 5) aids in identifying hotspot areas and times where/when intense rainstorms occur in comparison to low-intensity rainfall events. ED values exceeding 1 MJ ha^−1^ h^−1^ indicate the dominance of high intensity and short duration rainfall events, whereas values less than 1 MJ ha^−1^ h^−1^ indicate areas where low intensity rainfall events are prevalent. Knowledge of the temporal distribution of ED is required to identify periods when the risk of erosion is high because soil exposure occurs during months of high erosivity. Figure 5 shows that ED is high in southeastern North America during April, whereas large parts of South America, central Africa, and Australia experience high EDs from January to March. June to August is the period of high ED in southern Asia and northern Africa, including the Sahel (Fig. 5). Southeastern Asian counties typically experience high EDs for large parts of the year (Fig. 5). Northern parts of North America, northern Africa, northern Europe, and northern Asia are subject to relatively low EDs for large parts of the year. Overall, monthly ED values are relatively high from June to August in the tropical north, whereas EDs are high from January to April in the tropical south (Table S1). Foster et al.^46^ reported that EDs are strongly related to the average monthly 30-min rainfall intensity. Dabney et al.^47^ revealed that monthly EDs higher than 3 MJ ha^−1^ h^−1^ contribute substantially to the likelihood of high runoff. When monthly EDs are that high, regions are subject to high flooding and soil erosion risks.

**Figure 6:**
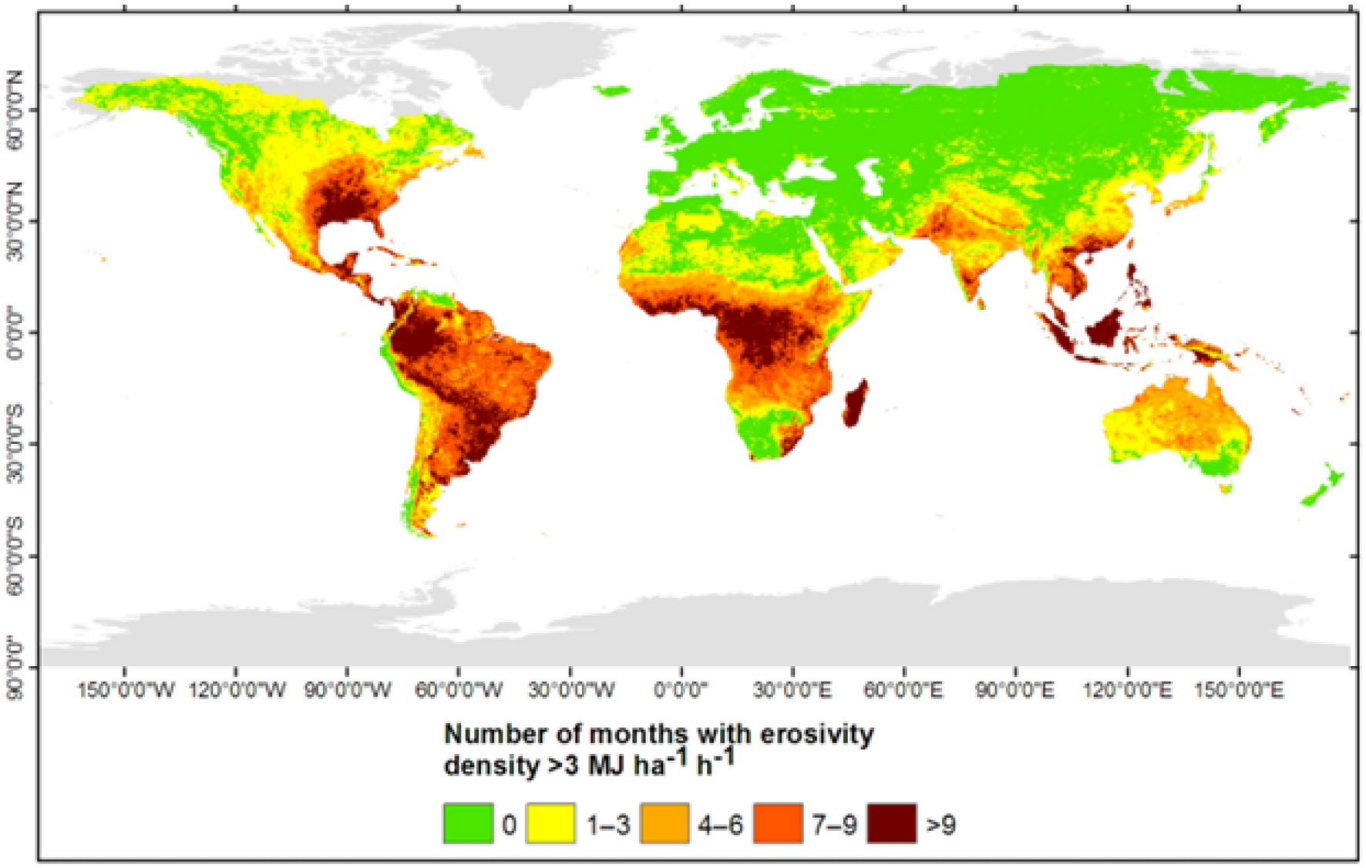
Map of the number of months per year with an erosivity density greater than 3 MJ ha^−1^ h^−1^, computed based on monthly erosivity density (Fig. 5). This map was produced using ArcGIS Pro version 3.2 (https://www.esrij.com/products/arcgis-pro/).

**Figure 7:**
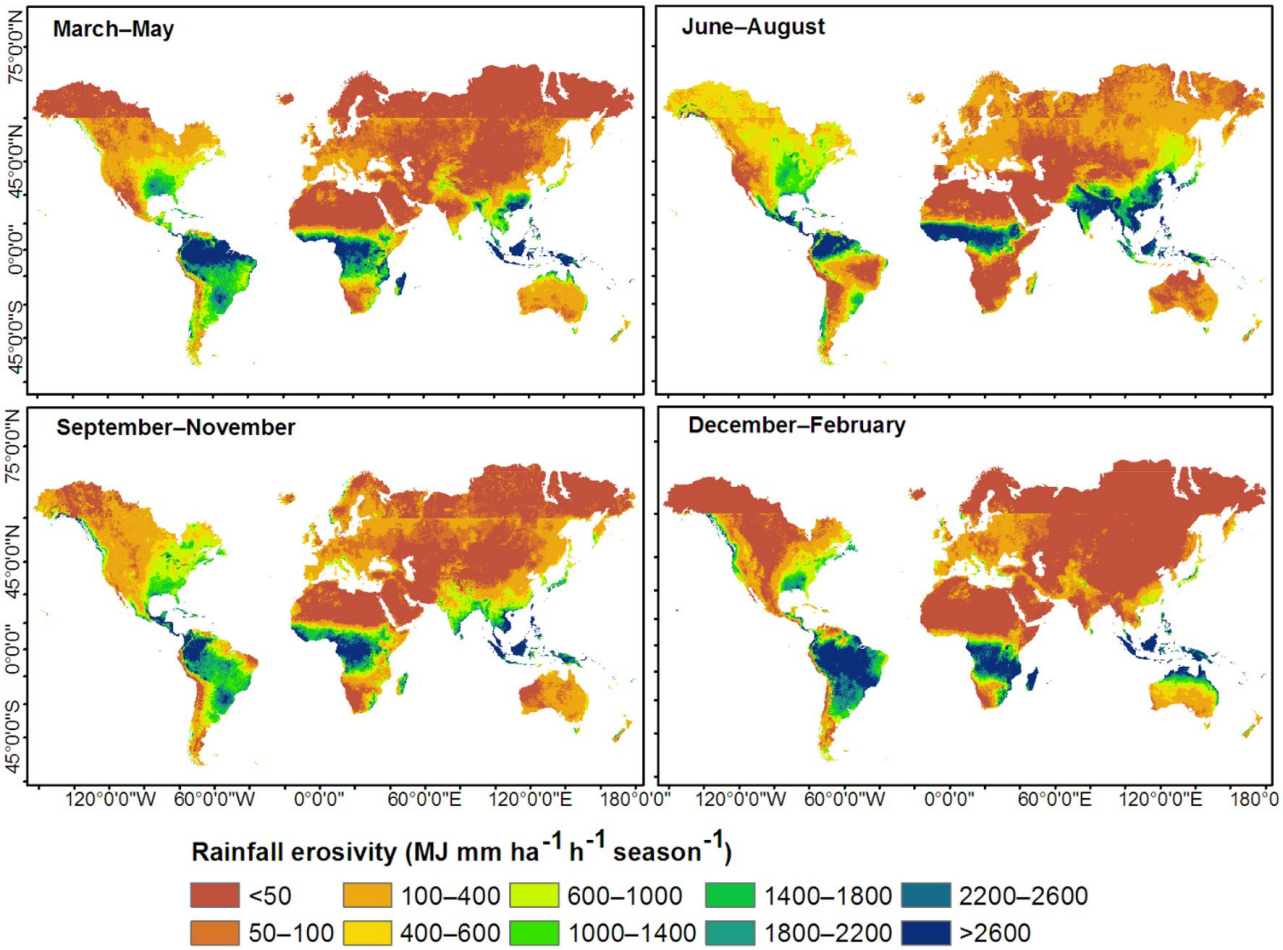
Spatial distribution of seasonal rainfall erosivity computed based on monthly rainfall erosivity (Fig. 2). This map was produced using ArcGIS Pro version 3.2 (https://www.esrij.com/products/arcgis-pro/).

Based on Dabney et al.^47^, we used very high monthly ED values (> 3 MJ ha^−1^ h^−1^) as thresholds to map regions that would be subject to more frequent erosive events on an annual basis (Fig. 6) and hence prone to potentially high soil erosion and/or landslides. The EDs are high during most months of the year (> 9 months yr^−1^) in the southeastern US, northwestern and southeastern North America, southern and southeastern Asia, central and western Africa, and Madagascar (Fig. 6). These regions are likely to be strongly affected by landslides and/or soil erosion. Large parts of India and Pakistan in Asia, Angola, Zambia, and Mozambique in Africa, and North America are subject to high monthly EDs for 7–9 months of the year (Fig. 6). The Sahel in Africa and central and northern Australia have 4–6 months of high monthly EDs. The northern part of North America, large swaths of Europe, central Asia, southern Australia, and northern and southern Africa are among the regions of less susceptibility (Fig. 7). Our result of ED-based mapping (Fig. 6) was in good agreement with studies of landslides and soil erosion at both the global and regional levels^1,48–50^. Parts of Southeast Asia, eastern and western Africa, and South America are experiencing high rates of soil erosion by water^1^. There have been reports of high-impact landslides in mountain ranges of Central and Southeast Asia as well as parts of North and South America^48,49^. In Africa, landslide-prone areas have been identified in central and western Africa and Madagascar^50^.

### Spatial distribution of seasonal rainfall erosivity

**Figure 8:**
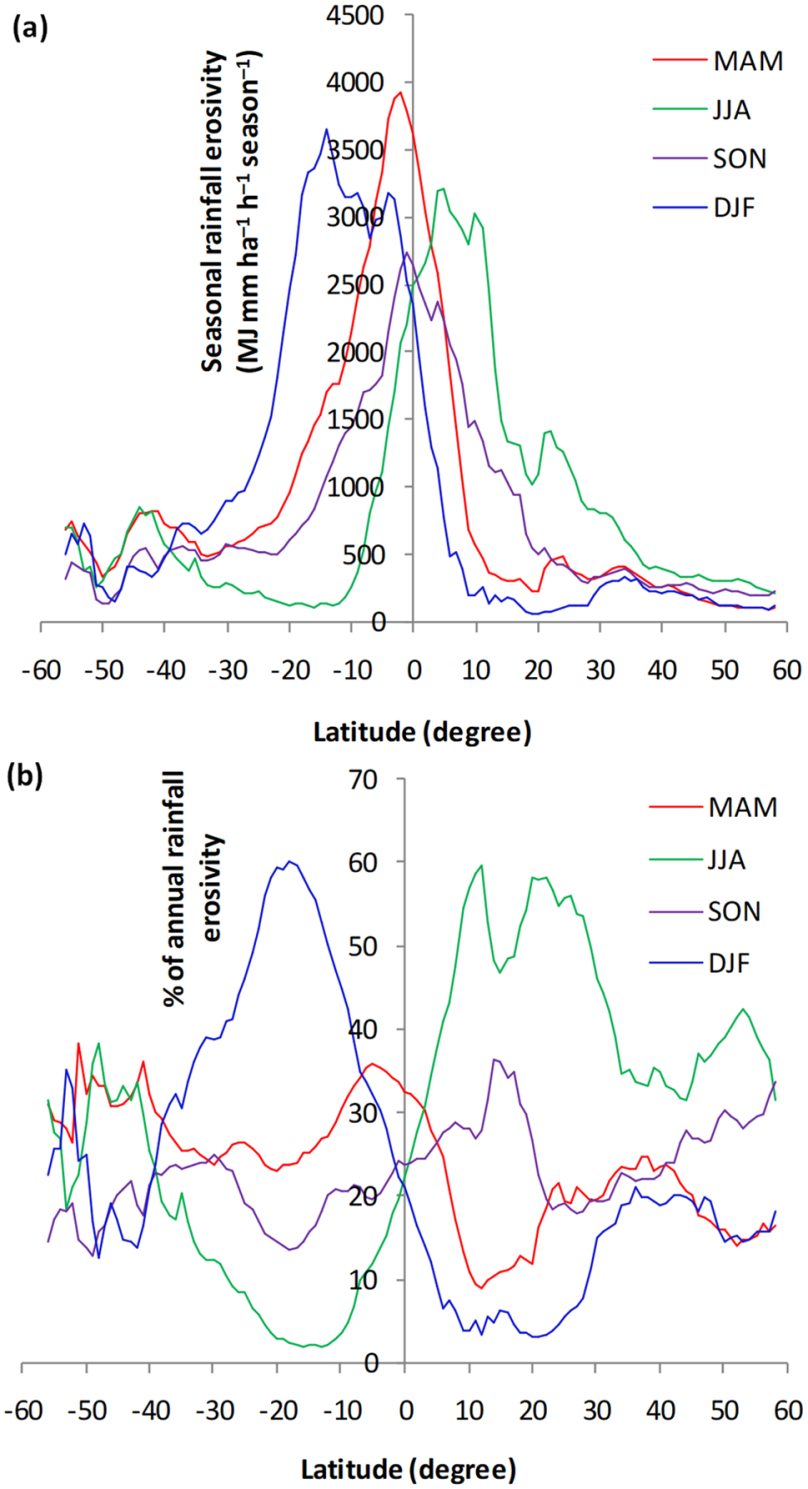
Latitudinal distribution of (**a**) seasonal rainfall erosivity and (**b**) the percent contribution of seasonal rainfall erosivity to annual rainfall erosivity, computed based on zonal averages at one-degree intervals between 60°S (–60°) and 60°N (60°). MAM, March–April–May; JJA, June–July–August; SON, September–October–November; DJF, December–January–February.

**Figure 9:**
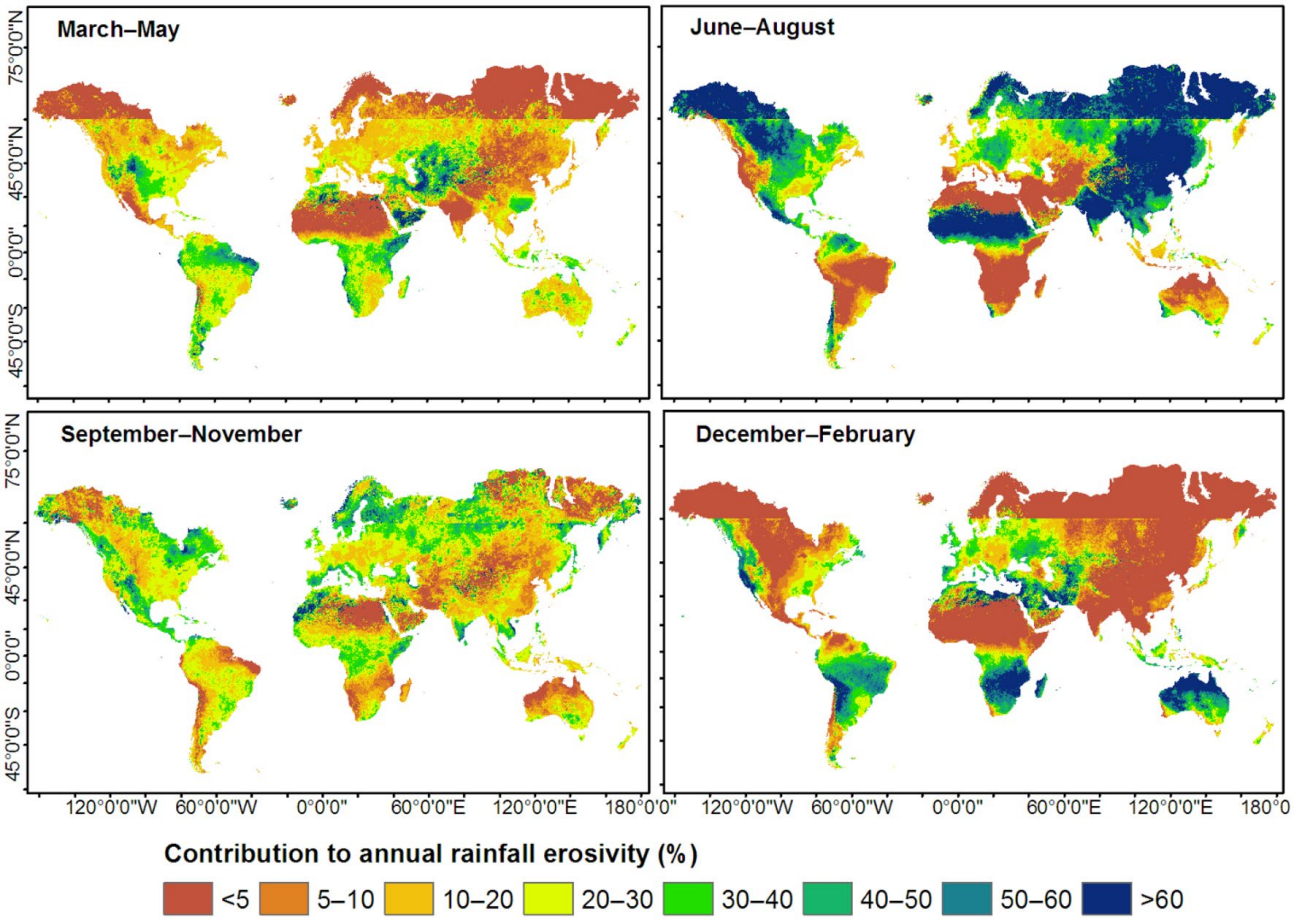
Percent contribution of seasonal rainfall erosivity to the total annual rainfall erosivity. This map was produced using ArcGIS Pro version 3.2 (https://www.esrij.com/products/arcgis-pro/).

June–August was the season of high erosivity (MJ mm ha^−1^ h^−1^ season^−1^) over sizable areas of North America and Europe, South America, southern Asia, and equatorial regions of Africa (Fig. 7). December–February was the season of high erosivity in large swaths of North America, southern Africa, and northern Australia (Fig. 7). Over the Northern Hemisphere, the annual cycle of rainfall erosivity peaked in June–August (~ 600 MJ mm ha^−1^ h^−1^ season^−1^, Table 2) and reached up to 3000 MJ mm ha^−1^ h^−1^ season^−1^ in some regions of the tropical north (Fig. 9a). Over the Southern Hemisphere, the cycle peaked in December–February (~ 2,000 MJ mm ha^−1^ h^−1^ season^−1^, Table 2) and could reach up to 3500 MJ mm ha^−1^ h^−1^ season^−1^ in some regions of the tropical south (Fig. 8a). December–February and June–August were the least erosive seasons in the Northern and Southern Hemispheres, respectively (Table 2). Rainfall erosivity was ~ 4 times higher during the most erosive seasons than the least erosive seasons (Table 2) for both the Northern and Southern Hemispheres. Over equatorial regions, seasonal rainfall erosivity peaked in March–May (~ 4,000 MJ mm ha^−1^ h^−1^ season^−1^, Fig. 8a). The seasonal distribution of rainfall erosivity (Fig. 7) followed the seasonality of rainfall^38,39^. The highest amounts of rainfall were received in June–August in the Northern Hemisphere and in December–February in the Southern Hemisphere.

**Table 2:** Distribution of mean seasonal rainfall erosivity (MJ mm ha^−1^ h^−1^ season^−1^).

Season	Tropical north	Tropical south	Northern Hemisphere	Southern Hemisphere	Global
MAM	1032	2013	279	1478	486
JJA	**2047**	546	**616**	469	**590**
SON	1316	1271	372	978	476
DJF	461	**2808**	167	**2046**	492

*MAM* March–April–May, *JJA* June–July–August, *SON* September–October–November, *DJF* December–January–February.

High values are shown in bold font.

The seasonal contribution of rainfall erosivity to the annual rainfall erosivity varied greatly over the Northern and Southern Hemispheres (Figs. 8b, 9). The differences were mainly related to latitude. Over large swaths of the Northern Hemisphere, the June–August season accounted for over 60% of the annual rainfall erosivity (Fig. 9). Over the Southern Hemisphere and the Middle East, December–February contributed more than 60% of the annual rainfall erosivity (Fig. 9). In the region of the horn of Africa (e.g., Somalia and southern Ethiopia), March–May made the highest contribution to the annual rainfall erosivity. The latitudinal profile (Fig. 8b) also showed that June–August and December–February were the main contributors to the annual rainfall erosivity in the Northern and Southern Hemispheres, respectively. At latitudes of 10–30°, i.e., in the tropics of both hemispheres, the contribution of seasonal rainfall erosivity varied strongly among seasons, whereas in equatorial and temperate regions, seasonal contributions were more-or-less equal throughout the year (Fig. 8b).

### Seasonal erosivity density

Different spatial patterns of ED were apparent during the four seasons (Fig. S4). The spatial pattern of seasonal ED (Fig. S4) followed the pattern of seasonal rainfall erosivity (Fig. 7). Globally, the ED was highest (2.1 MJ ha^−1^ h^−1^, Table S2) during June–August, when the rainfall intensity was relatively high. Seasonal ED peaked during June–August (~ 1.8 MJ mm ha^−1^ h^−1^, Table S2) over the Northern Hemisphere and during December–February and March–May (~ 4.3 MJ mm ha^−1^ h^−1^, Table S2) over the Southern Hemisphere. In both the tropical north and south, seasonal peak EDs were very high (> 4 MJ mm ha^−1^ h^−1^, Table S2). The seasonal EDs suggest that intense rainstorms dominantly occurred during June–August in the Northern Hemisphere, but were bimodally distributed between December–February and March–May in the Southern Hemisphere, in good agreement with the results of Zipser et al.^40^.

## Potential applications of the rainfall erosivity dataset

Rainfall erosivity data plays a pivotal role for modeling and managing soil loss by water erosion, providing valuable insights for sustainable land use and management. Besides, the rainfall erosivity dataset holds applicability across various domains, including water resources management, landslide risk assessment, flood risk assessment, and climate change impact assessment. Below, we briefly describe several potential regional- or country-level applications of the global monthly rainfall erosivity dataset:

- *Erosion hotspot identification:* integrating the rainfall erosivity data with other spatial datasets such as soil type, land cover, and topography can facilitate regional- or country-level identification of soil erosion hotspots—areas characterized by elevated soil erosion rates^4,10^. Identification of soil erosion hotspots can help prioritize where soil erosion control measures are urgently needed. This information guides targeted intervention strategies and resource allocation to address soil erosion challenges more effectively and support SDGs at regional or country scales.
- *Land use planning and management:* understanding the spatial and temporal variations in rainfall erosivity can inform land use planning and management strategies. The rainfall erosivity dataset allows for analyzing intra-annual variability of erosivity and identification of peak erosive periods with elevated soil erosion risks. By identifying periods of high rainfall erosivity, land managers can develop adaptive management strategies that account for seasonal erosivity patterns. These strategies may include implementing erosion control practices, adjusting land use practices, adjusting planting schedules, and formulating crop rotation scenarios tailored to specific months or seasons^17,20,21^.
- *Water resource management:* rainfall erosivity data can aid in water resource management by assessing the impact of soil erosion on water quality and quantity. Soil erosion can contribute to sedimentation in water bodies, affecting water supply, aquatic habitats, and ecosystem health. Analyzing rainfall erosivity data at a regional- or country-level can help identify areas where erosion-related sediment poses a threat to water resources^10,12^. Understanding erosivity patterns can thus inform strategies for watershed management and sediment control and support sustainable use and management of water resources.
- *Landslide risk assessment:* rainfall erosivity data helps identify periods of intense rainfall that can trigger landslides. High intensity (erosivity) values indicate rainfall events with a high potential to cause soil erosion and destabilize slopes, serving as early warning indicators for landslide risk^48^. Thus, rainfall erosivity data can play a crucial role in landslide risk assessment by providing useful insights into the erosive potential of rainfall events, which directly influence soil stability and slope integrity. The rainfall erosivity dataset can thus be used by landslide experts as a predictor (triggering factor) to improve landslide risk assessment^24^.
- *Flood risk assessment:* erosive rainfall events often contribute to flash flooding—associated with short and high-intensity (high erosivity) rainfall events—particularly in regions with steep terrain or poor drainage infrastructure. Spatio-temporal data of rainfall events (and rainfall erosivity) should be taken into account in the design of flash flood prediction and warning systems^24^. Rainfall erosivity data can play a vital role in enhancing flood risk assessment methodologies and supporting informed decision-making to mitigate the impacts of flooding.
- *Climate change impact assessment:* rainfall erosivity data can be used to assess the potential impacts of climate change on soil erosion patterns^6,7^. Changes in rainfall patterns, intensity, and frequency due to climate change can alter erosivity levels, affecting soil erosion rates. The rainfall erosivity data alongside climate projections can help anticipate future soil erosion trends, identify climate change hotspots, and develop adaptation strategies to minimize soil erosion risks under different climate change scenarios^6,7^.

## Potential sources of uncertainty

The density of GloREDa stations^23^ used to merge with our IMERG erosivity estimates was relatively low in some areas, specifically in Africa, South America, and parts of Asia. These low station densities may have affected the accuracy of our rainfall erosivity estimates. We used GloREDa^17,23^ as an independent dataset for comparison with the IMERG-based rainfall erosivity estimates. Nevertheless, when GPCC data were used to climatologically correct IMERG rainfall estimates, it is probable that some indirect use of GloREDa stations occurred, which could have biased our comparison. Nonetheless, the resulting bias was most likely insignificant because we made our comparison based on long-tern averaged data. In addition, it is highly likely that the IMERG-based rainfall estimations missed high intensity and short duration rainfall events because of the satellite’s low sampling frequency^51^, and hence we may have underestimated rainfall erosivity. The spatial resolution of the IMERG dataset was 0.1° × 0.1° (≈ 10 × 10 km), which could have limited our observations of local rainfall variability, for example, because of orographic effects or other microclimatic differences, and in turn could have affected our estimates of rainfall erosivity. PMW algorithms are limited in their ability to distinguish between clouds that are actually raining and surfaces that emit a microwave signature similar to that of precipitation^52^. Surfaces like sand and snow might thus be mistaken for precipitation signatures that are unique to PMW algorithms^52,53^. As a result, IMERG may overestimate the erosivity of rainfall in sandy desert and snowy regions^37^. Furthermore, IMERG uses rainfall estimates derived from inter-calibrated PMW sensors. Missing data in the PMW estimates are filled using geosynchronous IR-based rainfall estimates^26,54^. By morphing with the use of numerical model variables, the IMERG V06 enables coverage at high latitudes. However, because IR data is limited to 60°N–60°S, the advantages of morphing are diminished at high latitudes due to the significant differences between sensors over frozen surfaces^55^. This problem leads to greater uncertainties in the estimates of rainfall erosivity for regions poleward of 60°N and S. Moreover, IMERG dataset used in the present study covered the years 2001–2020, whereas the temporal coverage of most of the GloREDa stations was the years 2000–2010^23^. Such differences in the temporal coverage of the IMERG and GloREDa datasets may have caused a bias. The bias resulting from the difference in the temporal coverage may not have a marked effect since the integration of IMERG and GloREDa datasets was based on mean annual data, but this bias should be taken into account in future applications. Hence, there is an opportunity to enhance the accuracy of monthly rainfall erosivity estimates through using more homogenous data inputs and as more data become available from regions currently underrepresented, such as Africa, South America, and parts of Asia.

## Conclusions

The modeling of monthly and seasonal rainfall erosivities at a global scale using the IMERG and GloREDa datasets was an essential and novel component of this study. The results revealed that long-term mean monthly rainfall erosivity was highly dynamic within the timeframe of a year. Rainfall erosivity peaked in June–August (~ 200 MJ mm ha^−1^ h^−1^ month^−1^) over the Northern Hemisphere and in December–February (~ 700 MJ mm ha^−1^ h^−1^ month^−1^) over the Southern Hemisphere. Rainfall erosivity was about 4 times higher during those months than during the least erosive months: December–February and June–August in the Northern and Southern Hemispheres, respectively. The periods of peak rainfall erosivity contributed to over 60% of the annual rainfall erosivity over both hemispheres. The variations of the long-term mean monthly and seasonal rainfall erosivity between regions indicate the need for dynamic (monthly and seasonal) modeling of soil erosion. For instance, combining soil erosion factors (e.g., rainfall erosivity and cover management) that vary temporally could result in a more dynamic prediction of soil erosion within a year using the RUSLE. This approach could, in turn, help to identify the seasons and regions most susceptible to high soil erosion risks. ED maps can also help to identify the most erosive months because high monthly ED values correspond to high rainfall intensities and thus highly erosive events. Information on the variability of monthly rainfall erosivity can help land managers implement appropriate measures to mitigate erosion, such as proper selection of tillage practices and crop types to lessen the impact of highly erosive events on soils, improvement of soil cover through the use of crop residue or mulching, and stabilization of topsoil during months of high rainfall erosivity. Modeling the intra-annual (monthly and seasonal) variability of global rainfall erosivity is the first step to modeling monthly and seasonal soil loss by water erosion. Such modeling can be used to improve ongoing efforts to model soil erosion and develop soil conservation measures to combat land degradation caused by soil erosion, hence assisting in the achievement of the SDGs.

## Material and methods

### Data sources

*IMERG:* is a multi-satellite precipitation dataset available at temporal resolutions of 30-min, daily, and monthly and spatial resolution of ~ 0.1° × 0.1°^26^. The GPM mission is a joint NASA-JAXA project launched in 2014 that built on the success of the Tropical Rainfall Measuring Mission (TRMM) satellite launched in 1997. IMERG compensates for the limited sampling frequency of passive microwave (PMW) sensors by using a constellation of satellites and augments that information with geosynchronous infrared (IR)-based estimates of precipitation. TRMM and GPM core observatory serve as reference standards in their respective eras to integrate the IR-based and PMW-based rainfall estimates. Three marked improvements of the GPM core observatory over the TRMM satellite are: (i) the orbital inclination angle increased from 35° to 65° to cover more climate zones; (ii) Dual-Frequency Ku-band (13.6 GHz) and Ka-band (35.5 GHz) precipitation radars were included; and (iii) the GPM Microwave Imager has high-frequency channels (165.5 and 183.3 GHz) which significantly improve IMERG’s rainfall retrieval skills. Three different types of IMERG rainfall products are available for different user requirements as follows: near-real-time “Early” run (IMERG-Early), “Late” run (IMERG-Late), and “Final” run (IMERG-Final) with latencies of 4 h, 12 h, and 4 months, respectively. IMERG-Early and IMERG-Late products are targeted for near-real-time applications such as flood forecasting^54^. IMERG-Final is a research-quality product that undergone gauge adjustment using the Global Precipitation Climatology Center (GPCC) data. IMERG V06 provides high-latitude coverage for rainfall products in all runs. We used 20-years (2001–2020) of IMERG-Final 30-min rainfall intensity (mm h^–1^) data. The IMERG data were accessed from the GPM website (http://pmm.nasa.gov/data-access/downloads/gpm) over the full global domain (90°N–90°S). Huffman et al.^26^ and Hou et al.^54^ have provided detailed information on the IMERG algorithm and product description.

*GloREDa:* is a global dataset of rainfall erosivity developed using sub-hourly rainfall records from several countries across different geographic and climatic regions (Panagos et al., 2017). Temporal coverage of the GloREDa rainfall time series ranges from 5 to 52 years. The years 2000 to 2010 account for the majority of the data^23^. Geographical distribution of GloREDa stations varies among the continents. The largest contribution (48% of the total dataset) is from Europe while Asia and the Middle East provide 34% of the data. North America and the Caribbean contribute 146 GloREDa stations, with United States being the main contributor. The fewest GloREDa stations are in Africa and South America (5% of the total dataset). GloREDa stations in South America and Africa are clustered, but those in North America, Europe, and Oceania are geographically rather well distributed. Panagos et al.^23^ have reported that GloREDa stations accurately depict a wide range of rainfall erosivity values. We used data from 3,286 GloREDa stations that were located inside IMERG’s grid cells (0.1° × 0.1°) for land areas. Ballabio et al.^17^ have used GloREDa data to develop maps of monthly rainfall erosivity for Europe at ~ 0.01° × 0.01° resolution. We accessed the GloREDa station data^23^ and the mean monthly rainfall erosivity maps of Europe^17^ from the European Soil Data Centre (ESDAC, https://esdac.jrc.ec.europa.eu/).

*CHELSA:* Climatologies at High resolution for Earth’s Land Surface Areas (CHELSA) is a global mean monthly climate data produced at a spatial resolution of ~ 0.01° × 0.01° (Karger et al., 2017). ERA-Interim (http://www.ecmwf.int/en/research/climate-reanalysis/era-interim) is utilized to produce CHELSA by a statistical downscaling technique. The rainfall bias correction using Global Historical Climate Network (https://www.ncdc.noaa.gov/ghcnm/) and GPCC (https://www.dwd.de/EN/ourservices/gpcc/gpcc.html) data incorporates orographic effects^56^ (e.g., valley exposition, boundary layer height, and wind fields). In the recent version (CHELSA 1.2), the original statistical downscaling method has been enhanced to produce more reliable climatic datasets. Improvements have been made to the bias correction, which uses a 0.25° × 0.25° resolution GPCC data instead of a 0.5° × 0.5° resolution data which was used in the older version. The rainfall estimates provided by CHELSA are more accurate compared with rainfall data from other sources (such as WorldClim)^56^. The mean monthly rainfall data (1979–2013) used in this study was accessed from the CHELSA website^56^ (https://chelsa-climate.org/).

### Calculation of rainfall erosivity

For each IMERG grid cell (0.1° × 0.1°), rainfall erosivity was calculated as the product of the total storm kinetic energy (E) and maximum 30-min intensity (*I*_30_) of a rainfall event^57^. We used Eq. (1) as proposed by Brown and Foster^57^ to compute the specific kinetic energy of rainfall (*e*_*r*_) because this equation was also used by Panagos et al.^23^ to develop GloREDa:

1 $${e}_{r}=0.29\left[1-0.72{\text{exp}}\left(-0.05{I}_{r}\right)\right]$$

where *I*_*r*_ is rainfall intensity (mm h^−1^) during the *r*^*th*^ period.

For a given rainfall evet *j*, the 30-min erosivity index, EI_30_ (MJ mm ha^−1^ h^−1^), was computed as:

2 $${{\text{EI}}}_{30}={{\left(\sum_{r=1}^{t}{e}_{r}{v}_{r}\right)}_{j}\times \left({I}_{30}\right)}_{j}$$

where *e*_*r*_ is the specific kinetic energy of rainfall (MJ ha^−1^ mm^−1^) and *v*_*r*_ is the rainfall depth (mm) during the *r*^th^ of *t* periods.

The mean monthly rainfall erosivity (*R*_m_, MJ mm ha^−1^ h^−1^ month^−1^) was calculated as the mean of the accumulated event rainfall erosivities within a month:

3 $${R}_{{\text{m}}}=\frac{1}{n}\sum_{i=1}^{n}\sum_{j=1}^{k}{\left({{\text{EI}}}_{30}\right)}_{j}$$

where *k* is the number of erosive events in month *i* and* n* is the number of years covered by IMERG data.

The mean annual rainfall erosivity (*R*_S_, MJ mm ha^−1^ h^−1^ yr^−1^) was calculated as the sum of the mean monthly rainfall erosivities. We selected erosive events based on the event thresholds proposed by Renard et al.^9^.

Integrating rainfall erosivity estimations from IMERG and GloREDa.

We employed a residual-based merging scheme to integrate rainfall erosivity estimations from IMERG and GloREDa data using GWR^58^. The residual was calculated as the difference between the estimated amounts of mean annual erosivities from IMERG and GloREDa, *R*_S_ and *R*_G_, respectively. The rainfall erosivity estimated by IMERG integrated with GloREDa (*R*_F_) was computed as:

4 $${R}_{{\text{F}}}={R}_{{\text{S}}}+f\left({R}_{{\text{G}}}-{R}_{{\text{S}}}\right)$$

where *f(R*_G_* – R*_S_*)* denotes the residual to be bias-corrected in the IMERG rainfall erosivity estimates determined using GWR^58^ as:

5 $${f\left({R}_{{\text{G}}}-{R}_{{\text{S}}}\right)}_{i}={\beta }_{i0}+\sum_{k=1}^{n}{\beta }_{ik}{x}_{ik}+{\varepsilon }_{i}$$

where ${f\left({R}_{{\text{G}}}-{R}_{{\text{S}}}\right)}_{i}$ is the dependent variable (residual) at location* i*, *x*_*ik*_ is the *k*^th^ independent (explanatory) variable at location* i*, *β*_*ik*_ is the *k*^th^ regression coefficient at location* i*, *β*_*i*0_ is the intercept at location* i*, *n* is the number of predictor variables, and *ε*_*i*_ is the regression residual at location* i*.

Equation (5) can be written using matrix method as:

6 $$\mathbf{Y}=\mathbf{X}\otimes {\beta }{\prime}+\varepsilon$$

where the symbol ⊗ denotes logical multiplication; **X** is the matrix for the independent (predictor) variable, *β'* is the matrix for the local regression coefficients, and *ε* is the residual vector.

GWR estimates the coefficients at location* i* using a local weighted least squares regression as:

7 $${\widehat{\beta }}_{i}={\left({\mathbf{X}}^{{\text{T}}}{\mathbf{W}}_{i}\mathbf{X}\right)}^{-1}{\mathbf{X}}^{{\text{T}}}{\mathbf{W}}_{i}$$

where ${\widehat{\beta }}_{i}$ is the coefficient vector at location* i*, **X**^T^ is the transpose of the matrix **X**, and **W**_*i*_ is the spatial weight matrix at location* i*.

The estimates by ${\widehat{\beta }}_{i}$ for the observation at location* i* were computed as:

8 $${\widehat{y}}_{i}={\mathbf{X}}_{i}{\left({\mathbf{X}}^{{\text{T}}}{\mathbf{W}}_{i}\mathbf{X}\right)}^{-1}{\mathbf{X}}^{{\text{T}}}{\mathbf{W}}_{i}\mathbf{Y}$$

**Figure 10:**
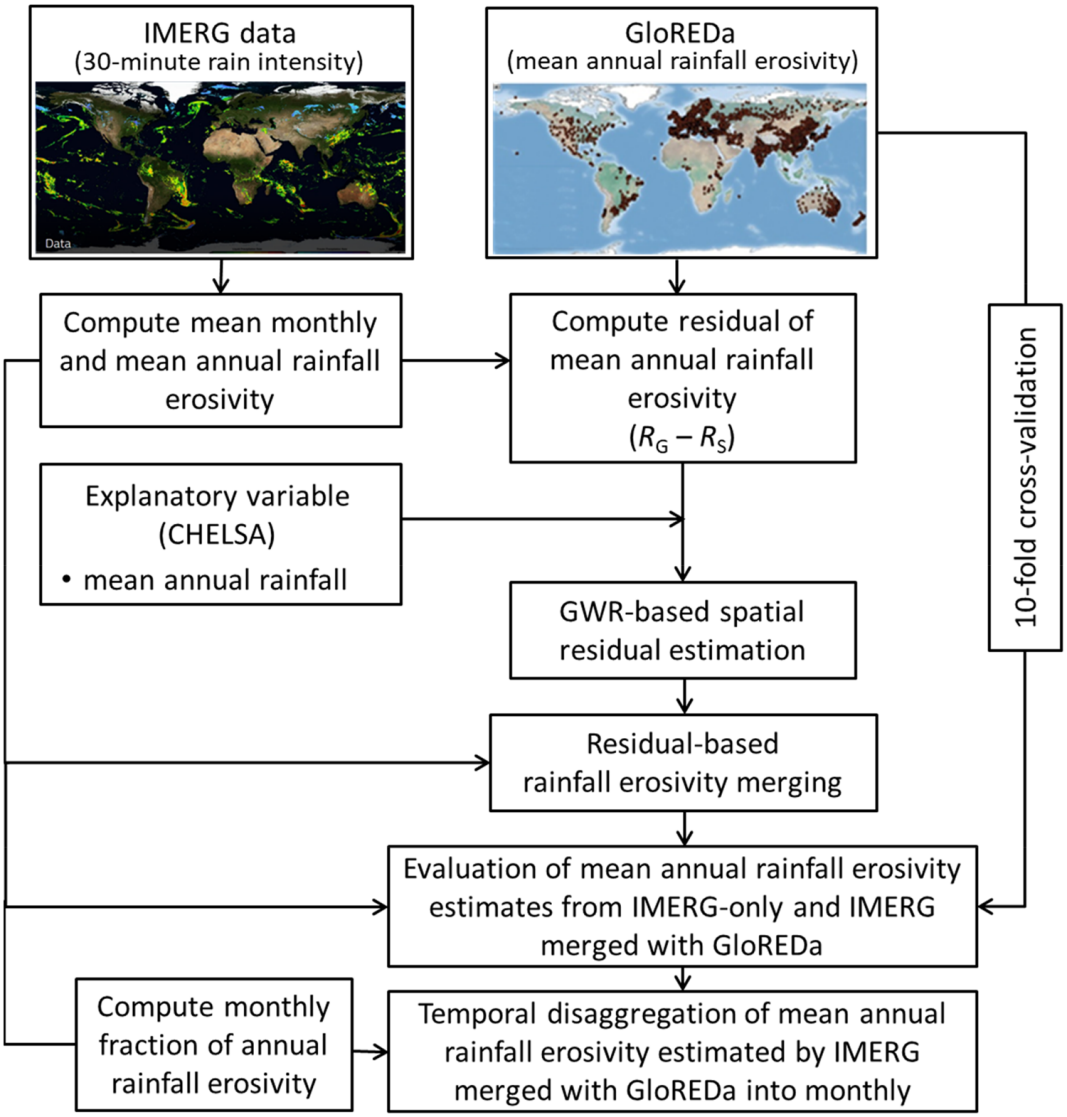
The general methodological framework of Geographically Weighted Regression (GWR)-based merging of the Integrated Multi-satellitE Retrievals for Global Precipitation Measurement (IMERG)-based mean annual rainfall erosivity estimates with gauge data from the Global Rainfall Erosivity Database (GloREDa; Panagos et al., 2017) using the mean annual rainfall from the Climatologies at High resolution for Earth’s Land Surface Areas (CHELSA) dataset as a predictor variable, and temporal disaggregation of the merged mean annual rainfall erosivity estimates into mean monthly rainfall erosivity estimates. The framework is modified from Fenta et al. (2023). *R*_G_, erosivity estimated from GloREDa gauge data; *R*_S_, erosivity estimated from IMERG satellite data.

To assign a geographical weighting function, a kernel type and bandwidth method should be specified. We used the adaptive Gaussian kernel function^59^ because it adapts well to the spatially variable distribution of GloREDa data (Fig. 10). The Akaike information criterion^60^ was applied to automatically determine the optimal bandwidth. Based on Eq. (7), the local regression coefficients were calculated for each IMERG grid cell. The local regression coefficients were substituted into Eq. (5) to compute the residual field at each IMERG grid cell. Finally, the mean annual rainfall erosivity estimated by IMERG merged with GloREDa was computed using Eq. (4).

The CHELSA’s mean annual rainfall (1979–2013) was used as an independent predictor variable for the GWR. Since elevation was utilized in CHELSA data to account for orographic influences^56^, it was not used as an independent predictor variable for the GWR. To improve the computational effectiveness of the GWR, both IMERG and CHELSA data were projected with a resolution of 10 × 10 km. A tenfold cross-validation was used to assess the efficacy of the GWR-based merging scheme using GloREDa data^23^. We used percent bias (PBIAS), Nash–Sutcliffe efficiency (NSE), and root mean square error (RMSE) to evaluate the accuracy of the GWR-based merging scheme. We also compared the monthly rainfall erosivities estimated by IMERG merged with GloREDa for Europe with monthly rainfall erosivity estimated by Ballabio et al.^17^ through spatial interpolation of the GloREDa data. The general methodological framework employed to integrate IMERG's rainfall erosivity estimations with GloREDa station data is shown in Fig. 10.

### Temporal disaggregation of merged annual rainfall erosivity estimates into monthly values

The merging of IMERG's rainfall erosivity estimations with GloREDa was done on an annual scale. However, to analyze intra-annual variability of rainfall erosivity, we used the fraction of monthly rainfall erosivities derived from the original IMERG data to disaggregate the mean annual rainfall erosivity estimated by integrating IMERG and GloREDa. This disaggregation procedure assumed that the monthly rainfall erosivity estimates from the original IMERG data captured the seasonal cycle of rainfall erosivity. We used the following steps to disaggregate rainfall erosivity from annual to monthly values: (i) the monthly fractions were estimated as the ratio of the monthly rainfall erosivity during the *i*th month to the annual rainfall erosivity estimated from the original IMERG data, and (ii) the merged annual rainfall erosivity estimates were multiplied by the corresponding monthly fractions to obtain the merged monthly rainfall erosivity estimates as:

9 $${\overline{R} }_{mi}=\frac{{R}_{{\text{m}}i}}{\sum_{i=1}^{n}{R}_{{\text{m}}i}}\times {R}_{{\text{F}}}$$

where ${\overline{R} }_{mi}$ is the merged mean monthly rainfall erosivity, *R*_m*i*_ is the mean monthly rainfall erosivity estimated from the original IMERG data for the *i*^*th*^ month, *n* is the number of months in a year (12), and *R*_*F*_ is the merged mean annual rainfall erosivity estimate from Eq. (4).

### Calculation of erosivity density

The erosivity density (ED) is the ratio of rainfall erosivity to rainfall amount^46^ (Eq. (10)). ED depends strongly on the number of high intensity rainfall events^46^. Small ED values (< 1 MJ ha^−1^ h^−1^) imply that rainfall erosivity is influenced mainly by the amount of monthly or seasonal rainfall, whereas high values show the prevalence of high intensity rainstorms with high kinetic energy relative to the observed amount of rainfall^46^. Also, ED indicates the influence of rainfall (short-lived, high-intensity events or large amounts of rainfall) on rainfall erosivity. In areas where high rainfall erosivity is particularly related to a few high intensity rainfall events (i.e., high ED values), the risk of soil erosion is high^47^. For a month or season *i*, ED is calculated as:

10 $${{\text{ED}}}_{i}=\frac{{R}_{i}}{{P}_{i}}$$

where ED_*i*_ is the monthly or seasonal erosivity density (MJ ha^−1^ h^−1^), *R*_*i*_ is the merged monthly or seasonal rainfall erosivity (MJ mm ha^−1^ h^−1^ month^−1^ or MJ mm ha^−1^ h^−1^ season^−1^), and *P*_*i*_ is the monthly or seasonal amount of rainfall (mm month^−1^ or mm season^−1^) from CHELSA.

### Supplementary Information

Supplementary Information.

## Data Availability

The mean annual, monthly, and seasonal rainfall erosivity data are available in the Mendeley Data, V1, https://doi.org/10.17632/brxhfgxppj.1. The IMERG rainfall data used in this study were downloaded from the GPM website (http://pmm.nasa.gov/data-access/downloads/gpm). The CHELSA rainfall data used in this study were accessed from https://chelsa-climate.org/.
