# Supplementary material for: An integrated modeling approach for estimating monthly global rainfall erosivity
Source: Sci Rep. 2024 Apr 8;14:8167. doi: 10.1038/s41598-024-59019-1 (PMC11001900; doi:10.1038/s41598-024-59019-1)
Supplement: Supplementary file 1 — Supplementary Information. [file 41598_2024_59019_MOESM1_ESM.pdf]

Supplementary Material

An integrated modeling approach for estimating monthly global rainfall erosivity

Ayele A. Fenta[1]*, Atsushi Tsunekawa[2], Nigussie Haregeweyn[1], Hiroshi Yasuda[3], Mitsuru Tsubo[2], Pasquale Borrelli[4,5], Takayuki Kawai[6], Ashebir S. Belay[7], Kindiye Ebabu[2,8], Mulatu L. Berihun[9,10], Dagnenet Sultan[9], Tadesual A. Setargie[2,9], Abdelrazek Elnashar[11], Arfan Arshad[12], and Panos Panagos[13]

[1]International Platform for Dryland Research and Education, Tottori University, Tottori 680-0001, Japan.
[2]Arid Land Research Center, Tottori University, 1390 Hamasaka, Tottori 680-0001, Japan.

[3]Organization for Educational Support and International Affairs, Tottori University, Koyama Minami 4-101, Tottori 680-8550, Japan.

[4]Department of Environmental Sciences, University of Basel, Basel, 4056, Switzerland.

[5]Department of Science, Roma Tre University, Rome, Italy.

[6]Graduate School of International Resource Sciences, Akita University, 1-1 Tegatagakuen-machi, Akita 010-8502, Japan.

[7]Department of Earth Science, Bahir Dar University, P.O. Box 79, Bahir Dar, Ethiopia.

[8]College of Agriculture and Environmental Sciences, Bahir Dar University, P.O. Box 1289, Bahir Dar, Ethiopia.

[9]Faculty of Civil and Water Resource Engineering, Bahir Dar Institute of Technology, Bahir Dar University, P.O. Box 26, Bahir Dar, Ethiopia.

[10]Tropical Research and Education Center, University of Florida, FL 33031, USA.

[11]Department of Natural Resources, Faculty of African Postgraduate Studies, Cairo University, Giza 12613, Egypt.

[12]Department of Biosystems and Agricultural Engineering, Oklahoma State University, Stillwater 74075, OK, USA.

[13]European Commission, Joint Research Centre (JRC), I-21027, Ispra (VA), Italy.

* Corresponding author

E-mail address: ayelealmaw@tottori-u.ac.jp, almawayele@yahoo.com (A.A. Fenta)

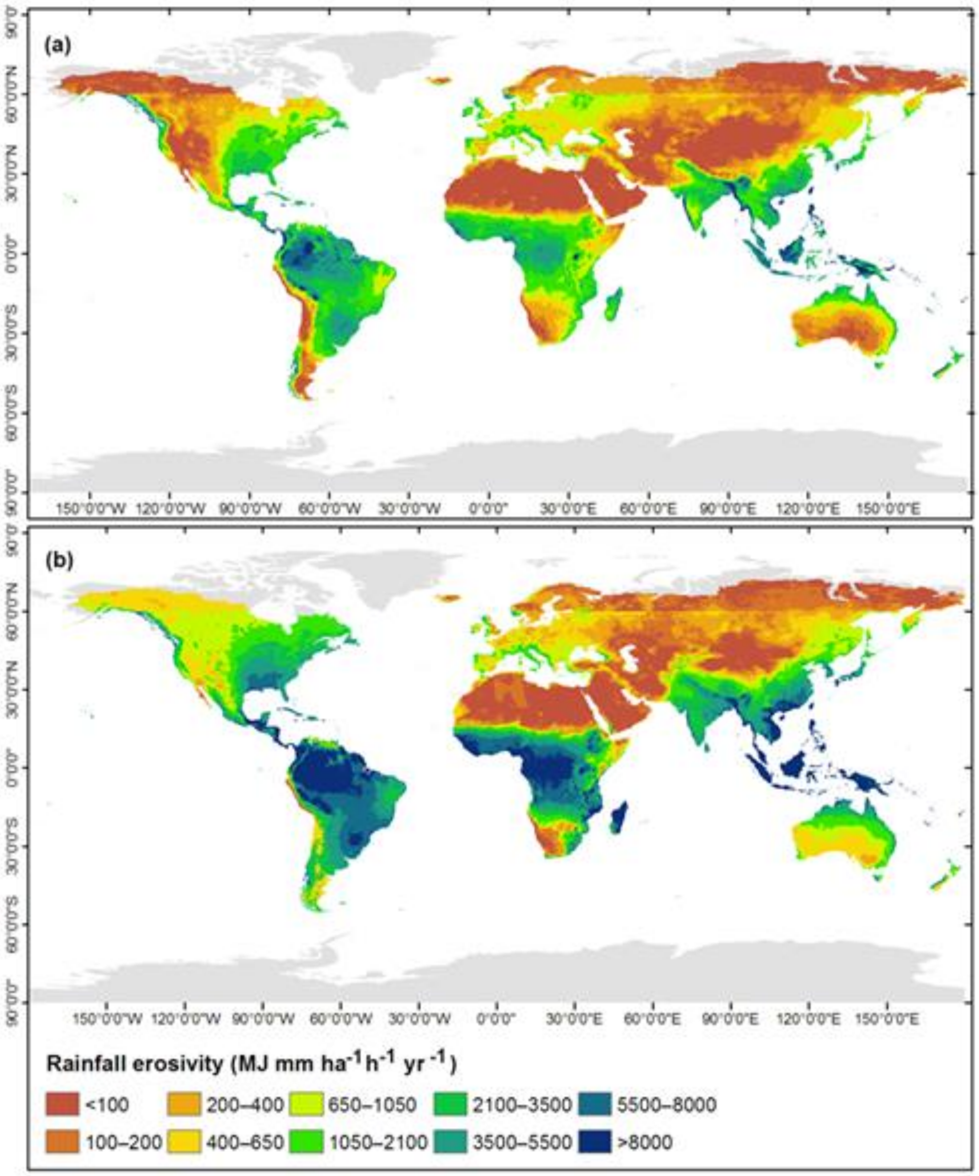

Fig. S1. Spatial distribution of mean annual global rainfall erosivity (2001–2020) based on (a) Integrated Multi-satellitE Retrievals for Global Precipitation Measurement (IMERG)-only and (b) IMERG merged with Global Rainfall Erosivity Database (GloREDa) station data. Erosivity classes correspond to quantiles of the IMERG merged with GloREDa. Source: Fenta et al. (2023). This map was produced using ArcGIS Pro version 3.2 (https://www.esrij.com/products/arcgis-pro/).

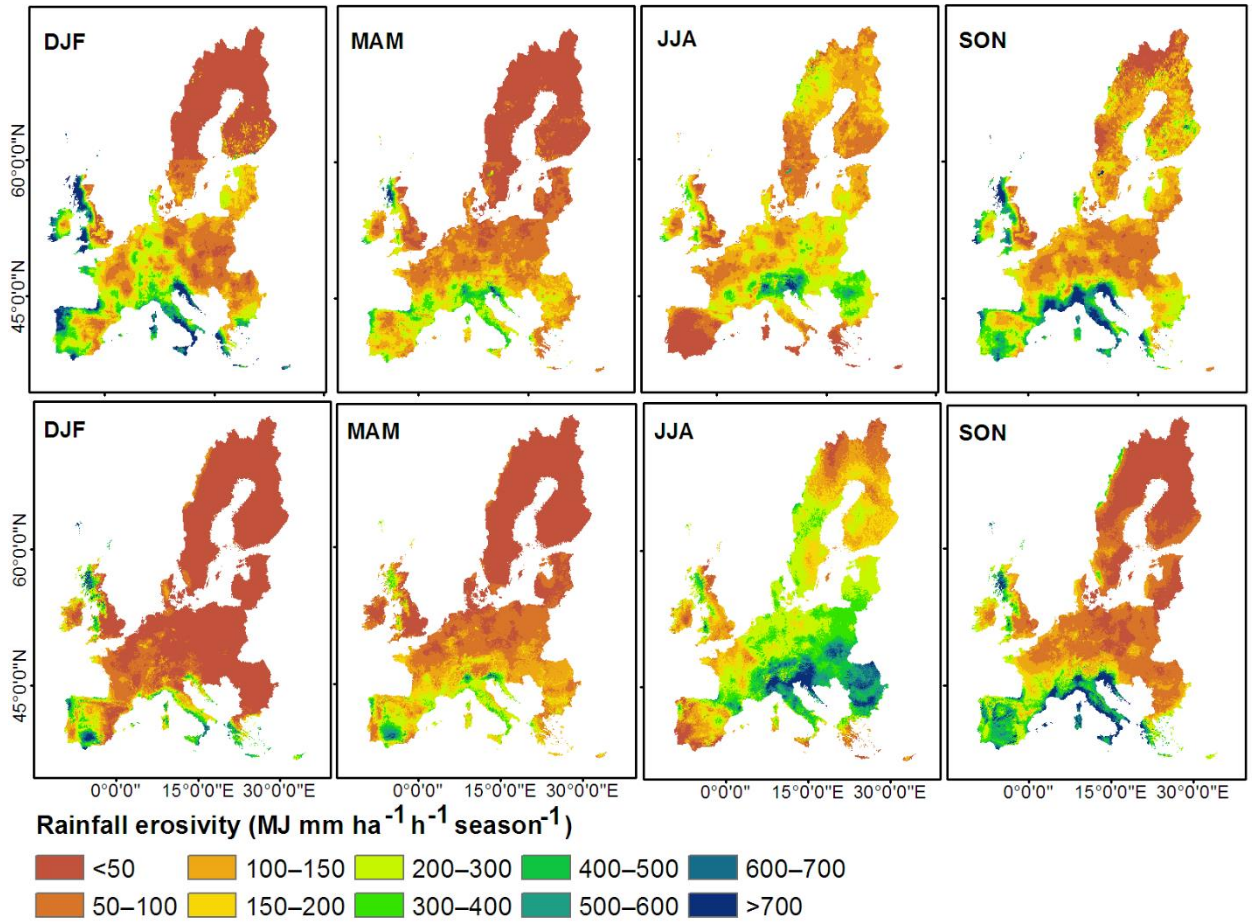

Fig. S2. Comparison between seasonal erosivity maps for Europe based on Integrated Multi-satellitE Retrievals for Global Precipitation Measurement (IMERG) merged with Global Rainfall Erosivity Database (GloREDa) (top panel) and GloREDa interpolated (Ballabio et al., 2017, bottom panel). This map was produced using ArcGIS Pro version 3.2 (https://www.esrij.com/products/arcgis-pro/).

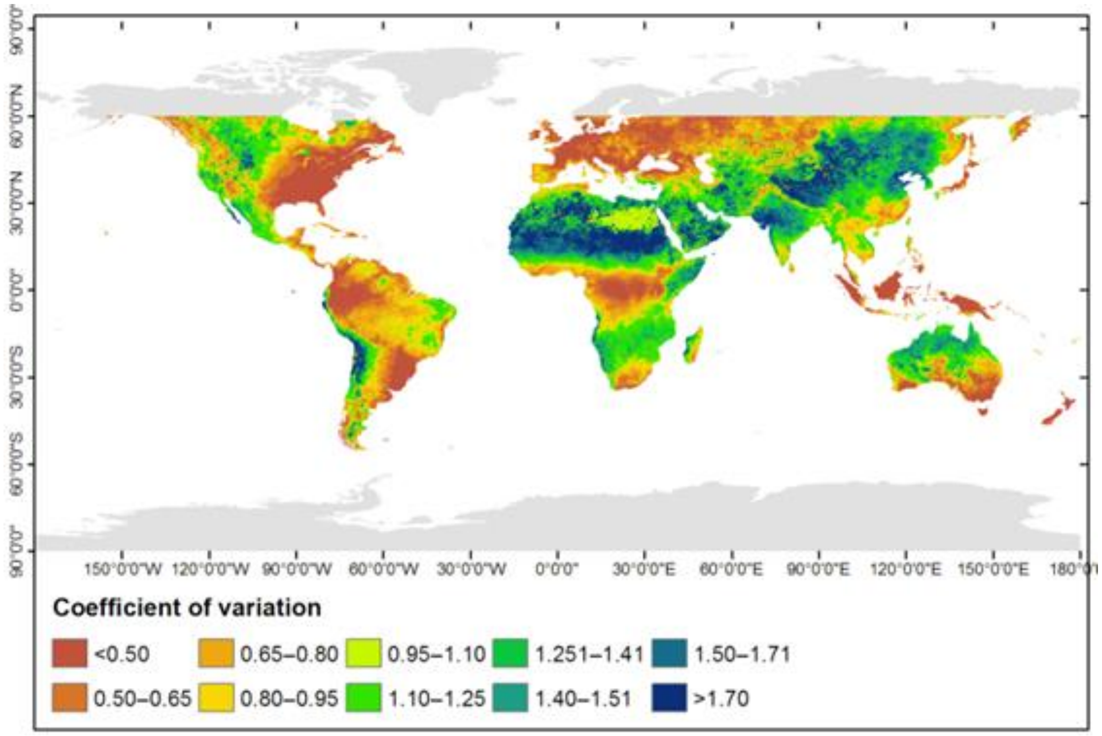

Fig. S3. Map of the Coefficient of Variation (CV) of the global monthly rainfall erosivity. This map was produced using ArcGIS Pro version 3.2 (https://www.esrij.com/products/arcgis-pro/).

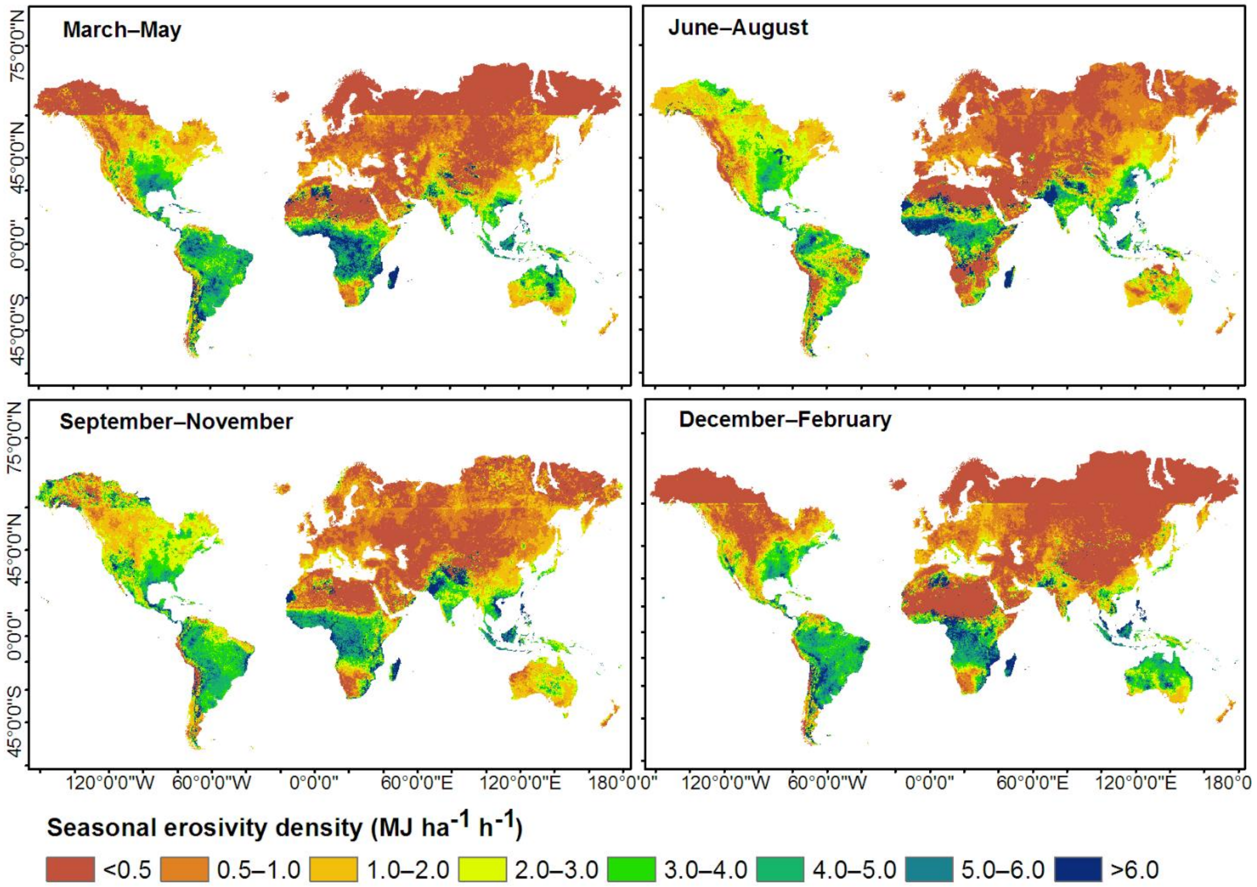

Fig. S4. Spatial distribution of seasonal erosivity density computed based on seasonal rainfall erosivity (Fig. 8) and mean seasonal rainfall from the Climatologies at High resolution for Earth's Land Surface Areas (CHELSA) dataset. This map was produced using ArcGIS Pro version 3.2 (https://www.esrij.com/products/arcgis-pro/).

Table S1. Distribution of mean monthly erosivity density (MJ ha^{-1} h^{-1}) over the northern and southern hemispheres. High values are shown in bold font.

Month	Tropical north	Tropical south	Northern hemisphere	Southern hemisphere	Global
January	2.25	**5.03**	0.99	**4.50**	1.60
February	2.51	**4.68**	0.94	**4.48**	1.55
March	2.39	**4.84**	1.07	**4.65**	1.69
April	2.50	**4.77**	1.15	**4.49**	1.73
May	3.68	4.04	1.36	3.53	1.73
June	**4.61**	4.09	**1.77**	3.63	**2.10**
July	**4.01**	3.38	**1.77**	2.91	**1.97**
August	**4.38**	3.06	**1.78**	2.76	**1.95**
September	2.78	2.54	1.56	2.37	1.62
October	2.79	3.72	1.49	3.36	1.81
November	2.93	4.56	1.19	4.02	1.68
December	2.53	4.48	1.02	4.08	1.55

Table S2. Distribution of mean seasonal erosivity density (MJ ha^{-1} h^{-1}) over the northern and southern hemispheres. *MAM:* March–April–May; *JJA:* June–July–August; *SON:* September–October–November; *DJF:* December–January–February. High values are shown in bold font.

Season	Tropical north	Tropical south	Northern hemisphere	Southern hemisphere	Global
MAM	2.88	**4.74**	1.23	**4.34**	1.77
JJA	**4.37**	3.80	**1.78**	3.30	**2.05**
SON	3.08	3.72	1.59	3.32	1.88
DJF	2.71	**4.75**	1.05	**4.34**	1.62

Reference

Ballabio, C., Borrelli, P., Spinoni, J., Meusburger, K., Michaelides, S., Beguería, S., Klik, A., Petan, S., Janeček, M., Olsen, P. and Aalto, J., 2017. Mapping monthly rainfall erosivity in Europe. Sci. Total Environ. **579**, 1298–1315.

Fenta, A.A., Tsunekawa, A., Haregeweyn, N., Yasuda, H., Tsubo, M., Kawai, T., Ebabu, K., Berihun, M.L., Belay, A.S., Sultan, D., Elnashar, A., Borrelli, P. and Panagos, P. 2023. Improving satellite-based global rainfall erosivity estimates through merging with gauge data. J. Hydrol. **620**, 129555.

Panagos, P., Borrelli, P., Meusburger, K., Yu, B., Klik, A., Jae Lim, K., Yang, J.E., Ni, J., Miao, C., Chattopadhyay, N. and Sadeghi, S.H., 2017. Global rainfall erosivity assessment based on high-temporal resolution rainfall records. Sci. Rep. **7**, 1–12.
